# Remarkable Case of Somnambulism

**Published:** 1869-11

**Authors:** 


### Original Communications.

ARTICLE I.—*Remarkable Case of Somnambulism.* By J. A. A.

IT was my fortune during a series of years to have under direct observation, a case of somnambulism, in some respects more remarkable than any other upon record. The subject was a family relative and private medical student of my own. He attended his first course of medical lectures at the old Indiana Medical College at Laporte, in 1849, and subsequently graduated at the Medical Department of the Michigan University at Ann Arbor, in 1852. He entered upon the practice of medicine at Holland, Mich., soon after, where he continued until a little after the commencement of the war, when he became connected with the army as a contract surgeon, and was in active service until its close. For some little time after, he sought restoration of his shattered health by rest, but unfortunately, whilst yet feeble, he was prostrated by typhoid pneumonia, to which he succumbed in a few days. He was widely known, and very many persons are familiar with some of the facts about to be detailed.

The first time he was ever known to walk in his sleep, was in the spring of 1847, and the first attempt was an unfortunate one, as he fell into a stairway which was unprotected by balusters, injuring himself considerably, although fracturing no bones. He had risen and dressed himself, but when awakened by his fall was utterly ignorant of his whereabouts. I saw him a few minutes afterwards and found nothing unusual about him, but he remarked that he had not felt quite well when he went to bed. For some months subsequently he would now and then get up, dress himself and go about the house without any apparent object, and usually after a while return to his bed voluntarily, awaking in the morning with not the slightest recollection even of a dream.

My young friend was an enthusiast in music, and a very respectable amateur. About the summer of 1847, a somewhat dilapidated bass viol, which was a kind of heir-loom in the family, was brought into the house, and he devoted spare moments to learning how to play upon it. Unfortunately, the antiquity of the instrument had told upon its keys, and unless they were wetted at each time of use, it would not remain in tune. He was determined, however, to command its notes, and succeeded. His somnambulic walks, thereafter, led him from his chamber to the parlor, and to the bass viol, and the family would be awakened in the small hours, by the inevitable tuning up prelude, mingled with slipping of the old keys, and quiet objurgations upon his part. Sometimes the bridge would fall down when the keys slipped, and sometimes a string would snap or escape from the keys, nevertheless he would persevere, repair damages, tune up, and then execute all varieties of music of which the machine was capable, not unfrequently accompanying it with his voice. All this would be done in total darkness. When any one entered the room with a light, he took not the least notice, although when spoken to he would reply in monosyllables or with considerable asperity. His face was usually flushed, although sometimes pale — the features immobile and passive, the eye open, pupil dilated, the surface glazed, and the lids apparently motionless. The extremities warm and the pulse full, frequent and soft. Very often the skin would be bathed with free perspiration. Remarkably sensitive to titillations when awake, there seemed total absence of reflex movements from this cause, whilst in the somnambulic state.

As he extended his acquaintance with music and musical instruments, his feats became wonderful. Whilst in attendance upon the Medical College at Laporte, the household looked forward with high anticipations to the hours when his skillful touch of the melodeon would wake them. He had a voice of the purest tone and very considerable compass, in fact, of rare sweetness. I am enabled to say from a multitude of observations, that he played with a precision and skill while asleep, that he could not approximate while awake. Beside this, he would execute music which he had heard, perhaps, but once, the evening previous or after a long interval — no note of which he could recall in his waking moments. His memory here seemed wonderfully exalted. If interrupted, he was irritable in the extreme, but would go on with his music exactly from the point of interruption.

Among the numberless exhibitions of his somnambulism, I have time only to notice a few of the most striking.

Whilst attending lectures at Ann Arbor, where I was then lecturing on Physiology, I requested his assistance in enlarging some of the drawings illustrative of minute anatomy and histology, for use in class demonstrations. He entered into the work with great zeal, and proved very expert and rapid in execution. One evening, previous to the day on which I was about to lecture on the kidney, I wished the cuts in Carpenter's Physiology, illustrating the tubular arrangement, etc., were ready. He had an engagement for the evening, but said he would try and prepare them in the morning. During the night he rose, dressed himself, played a few tunes on the guitar, part of the time singing (and, by the way, the guitar was about as dilapidated as the bass viol before noticed, and he had to knot one or two of the strings first), and then arranged the drawing paper, prepared his India ink and brushes, took the parallels and pencils and laid off the spaces, and worked for half an hour or more rapidly and perfectly, nearly completing the figures on pp. 596 and 597 of Carpenter's Principles, in the edition of 1853. His were taken from a previous edition not now in my possession. These drawings are now in the series used for illustration in Rush Medical College. Although we had a light in the room while watching him, he went on with his work entirely regardless of it. Before completing the work, he went to bed and slept until the usual hour in the morning, when at the breakfast he asked if he had been up in the night, as he had *dreamed* that he had. This was the only time he ever remembered even dreaming about being up or occupied in anything. He had by this time become so fully aware of his habits, that nothing of the sort astonished him. Shortly after this he went to spend the night with a fellow student, but a little after midnight he rose, dressed himself, and went out, followed by the other gentleman, walked down to the Exchange Hotel, where there were a number of his acquaintances and others waiting for a train of cars due at that time. Some one rallied him on his being out so late, but being cautioned by his companion, they did not attempt to awake him, but watched his movements. On being invited, he took a glass of ale, and then said he would only have time to go home and get his dinner before the afternoon lecture hour. He walked with his friend to our door, and was indignant to find it locked. His room mate (a cousin) admitted him and awakened myself and wife. He asked if dinner was ready, and seemed astonished that it was not; then said he would get a drink of water and be off, "for old D. (one of the faculty) would be mad if he was late." I told him he had plenty of time and he need not be in a hurry. He then walked into the kitchen, drank a tumblerful of water, and looking up to the clock, although it was totally dark, remarked the time, and started for the front door. I then told him that I was not feeling well, was pretty blue, and wished he would sit down and play euchre with us. This seemed to please him, and he took off his overcoat and said he had as lief play until "old D." was through lecturing, as to go.

His cousin sat down at the table with us, and we played "three-handed (cut-throat) euchre." He paid not the slightest attention to us, although we passed the cards backwards and forwards between us, exchanging hands, and everything we could do to attract his attention. He dealt the cards in his turn, correctly, and played "according to Hoyle." In one hand, spades were trumps, and he held the jack of clubs. Clubs being led, he first threw down this jack, then quickly picked it up, saying, "I forgot that was the left bower." It is somewhat humiliating to record that, notwithstanding our tricks and devices, he beat us in the game.

On its conclusion, he got up hastily and insisted upon going to the college. We only prevented him this time, by throwing water in his face — the only method, by the way, in which we could awake him without great violence. Pungent odors, ammonia, camphor, etc., he seemed to disregard, or merely pushed away the object.

On regaining consciousness, he always appeared like one stunned, or suffering from a severe shock. The influence upon the pulse and nervous system was always so severe, that we never awaked him at these times if we could avoid it.

Whenever a little out of health, as from trifling attacks of indigestion, or after watching with the sick, or fatigue, he would be sure to be up and doing something notable in the somnambulic state.

One of the most remarkable of his exploits occurred several years after the incident just given. I think it was in 1860 or 1861.

He gave me the particulars himself, and I have had the necessary concurrent evidence from others. The circumstances were so extraordinary, that they almost caused him to determine never to practice medicine again.

In the rounds of his practice he had a patient, about whom he was very anxious. It was in the coldest winter weather, and the residence of the patient was about two miles distant. Visiting him early in the evening, he found him in a state so unsatisfactory, that he informed the family that if he did not find him better the next visit, he should change the medicine entirely. On rising the next morning, he went to the barn to put his horse to the cutter for an early start. He was a little puzzled at finding things somewhat misplaced, but supposed some person had been in the stable in search of a missing article. On visiting the patient, he was gratified to find a marked improvement. He inquired when the improvement commenced, and was answered, "Immediately after taking the powders which he had given in the night." The truth flashed upon him at once, but concealing his emotion, he inquired, with as careless an air as he could assume, "About what time was it when I was here?" They replied, "Between two and three o'clock." This proved to have been the case, as he was afterwards told by the family where he boarded. He had been giving the patient some fluid medicine, which he ordered discontinued, and then put up several powders, such as he had concluded upon the night previous, combining them as usual, and administering the first one himself.

The alarm of my poor friend, at the possible consequences of a similar act in the future, may well be conceived.

The considerable length to which this sketch has already extended, must prevent comment on the peculiar features of this case, which must, as before suggested, be considered one of the most remarkable of record. I could detail a large number of equally striking facts in his history, with experiments instituted as to the physical and psychological condition, did time and space permit.

It illustrated most forcibly the automatic action of certain of the mental faculties, irrespective of *mental* consciousness.

There is a physical consciousness which is something else than a mental consciousness. There is a physical volition which is something else than a mental volition.

All the so-called mental acts, may be carried on automatically, without mental consciousness.

The "unconscious cerebration" of Carpenter, is only an illustration of this automatic action.

In conclusion, it is not out of place to remark, that my friend was of a high order of intelligence, and never could be made a "subject," or a "medium," by the disciples of Mesmer.

